# Supplementary material for: The Risk of Colorectal Polyps after Weight Loss Therapy Versus Obesity: A Propensity-Matched Nationwide Cohort Study
Source: Cancers (Basel). 2023 Sep 30;15(19):4820. doi: 10.3390/cancers15194820 (PMC10571780; doi:10.3390/cancers15194820)

Table S1. Demographics of eligible cohort prior to matching.

Variable		Colonoscopy pre-BRS (n=11112)	Colonoscopy post-BRS (n=9555)	Colonoscopy pre-SO (n=117802)	Colonoscopy post-SO (n=142948)	*p*-value
**Age at time of colonoscopy**		52 [48-57]	52 [49-59]	55 [51-60]	56 [51-62]	<0.001
**Sex**						<0.001
**Male**		2713 (24.4)	2164 (22.7)	47038 (39.9)	55030 (38.5)	
**Female**		8399 (75.6)	7391 (77.4)	70764 (60.1)	87918 (61.5)	
**Charlson comorbidity index**		3 [2-4]	2 [1-3]	3 [2-4]	2 [1-4]	<0.001
**Years from pre-BRS or pre-SO colonoscopy to index visit date***	**Median [IQR]**	0.8 [0.3-1.8]	n/a	1.3 [0.5-2.4]	n/a	<0.001
	**Range**	0.003-6.3	n/a	0.003-6.5	n/a	
**Years from index visit date to post-BRS or post-SO colonoscopy**	**Median [IQR]**	n/a	2.7 [1.8-4.2]	n/a	2.3 [1.6-3.6]	<0.001
	**Range**	n/a	1.0-8.5	n/a	1.0-8.7	
**Screening colonoscopy indication**		6170 (55.5)	5803 (60.7)	64868 (55.1)	86012 (60.2)	<0.001
**Alcohol use**		133 (1.2)	48 (0.5)	1544 (1.3)	1001 (0.7)	<0.001
**Tobacco use**		1751 (15.8)	1026 (10.7)	16009 (13.6)	12244 (8.6)	<0.001

Table S2: Polyp rates in the pre-matching cohort

**All patients**				
**Variable**	**Colonoscopy pre-BRS (n=11112)**	**Colonoscopy post-BRS (n=9555)**	**Colonoscopy pre-SO (n=117802)**	**Colonoscopy post-SO (n=142948)**
**Colorectal polyps**	5250 (47.3)	4389 (45.9)	61067 (51.8)	81382 (56.9)
**Rectal polyps**	1260 (11.3)	753 (7.9)	14258 (12.1)	13264 (9.3)
**Males**				
	**Colonoscopy pre-BRS (n=2713)**	**Colonoscopy post-BRS (n=2164)**	**Colonoscopy pre-SO (n=47038)**	**Colonoscopy post-SO (n=55030)**
**Colorectal polyps**	1533 (56.5)	1201 (55.5)	28335 (60.2)	35258 (64.1)
**Rectal polyps**	393 (14.5)	215 (9.9)	6744 (14.3)	6083 (11.1)
**Females**				
	**Colonoscopy pre-BRS (n=8399)**	**Colonoscopy post-BRS (n=7391)**	**Colonoscopy pre-SO (n=70764)**	**Colonoscopy post-SO (n=87918)**
**Colorectal polyps**	3717 (44.3)	3188 (43.1)	32732 (46.3)	46124 (52.5)
**Rectal polyps**	867 (10.3)	538 (7.3)	7514 (10.6)	7181 (8.2)

Table S3: The full pre-matched cohort. Comparison of colorectal/ rectal polyps' unadjusted odds ratios by sex pre- and post-BRS, pre- and post-SO

**Unadjusted OR (95% CI) Post- vs. Pre- colonoscopy**		**Males**	**Females**
**Colorectal polyps**	BRS	0.96 (0.86-1.08)	0.96 (0.90-1.02)
	SO	1.18 (1.15-1.21)	1.28 (1.26-1.31)
	BRS vs SO	0.82 (0.73-0.92)	0.75 (0.70-0.80)
**Rectal polyps**	BRS	0.65 (0.55-0.78)	0.68 (0.61-0.76)
	SO	0.74 (0.72-0.77)	0.75 (0.72-0.77)
	BRS vs SO	0.88 (0.73-1.05)	0.91 (0.81-1.02)

Table S4: The full pre-matched cohort. Comparison of rectal polyp odds ratios by sex pre- and post-bariatric surgery, pre- and post-severe obesity in full cohort prior to matching

**Post- vs. Pre- colonoscopy**		**Males**	**Females**
**Adjusted* OR (95% CI)**	BRS	0.92 (0.77-1.11)	1.00 (0.89-1.13)
	SO	1.08 (1.03-1.13)	1.11 (1.06-1.15)
	BRS vs SO	0.86 (0.71-1.03)	0.91 (0.81-1.02)

*Models adjusted for age at colonoscopy, tobacco use, alcohol use, Charlson comorbidity index, screening colonoscopy, date of colonoscopy (before/after Oct. 1, 2015)

Table S5: The propensity-matched analysis. Comparison of colorectal/ rectal polyps' unadjusted odds ratios by sex pre- and post-BRS, pre- and post-SO

**Unadjusted OR (95% CI) Post- vs. Pre- colonoscopy**		**Males**	**Females**
**Colorectal polyps**	BRS	0.80 (0.63-1.02)	0.99 (0.87-1.13)
	SO	1.32 (1.03-1.70)	1.26 (1.11-1.44)
	BRS vs SO	0.61 (0.43-0.86)	0.79 (0.66-0.94)
**Rectal polyps**	BRS	0.67 (0.45-0.99)	1.05 (0.83-1.34)
	SO	1.08 (0.73-1.61)	0.85 (0.68-1.08)
	BRS vs SO	0.62 (0.35-1.08)	1.23 (0.88-1.73)

BRS: Bariatric Surgery; SO: Severe Obesity

Table S6: The propensity-matched cohort. Rectal polyps' rates by group and sex - the propensity-matched analysis.

	Pre-BRS (n=2420)	Post-BRS (n=2420)	Pre-SO (n=2420)	Post-SO (n=2420)
**All patients**				
**Rectal polyps**	8.5%	7.9%	8.9%	8.2%
**Males**				
**Rectal polyps**	12.5%	8.7%	10.0%	10.8%
**Females**				
**Rectal polyps**	7.4%	7.7%	8.6%	7.5%

BRS: Bariatric Surgery; SO: Severe Obesity

Table S7: The propensity-matched cohort. Diabetes at index visit date, defined as date of bariatric surgery. Diabetes medications' cessation or new usage at time of colonoscopy.

**Variable**	**BRS Males (n=530)**	**BRS Females (n=1890)**
Diabetes at index	305 (57.5)	775 (41.0)
Diabetes medications at index	237 (44.7)	568 (30.1)
Cessation of diabetes medications at post-index colonoscopy	138/237 (58.2)	355/568 (62.5)
Began diabetes medication after index	9/293 (3.1)	44/1322 (3.3)

Results are presented as count (percentage)

Figure S1: Flowchart of 1:1:1:1 propensity matching algorithm (LPS SD: Logit of propensity score standardized difference)

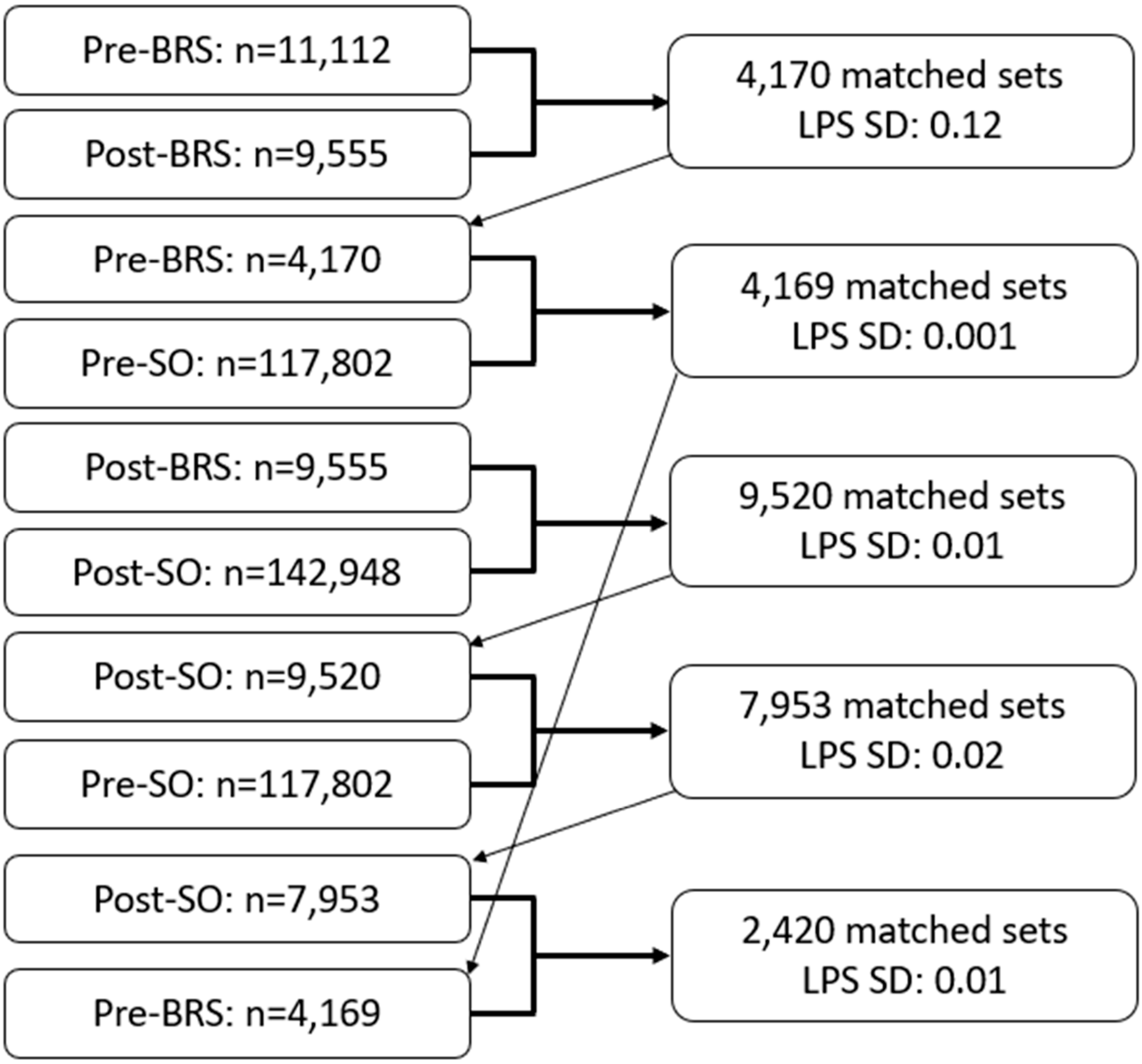

Supplement: Supplementary file 1 [file cancers-15-04820-s001.zip › cancers-2614856-supplementary.pdf]
